# Association of bone mineral density with bone texture attributes extracted using routine magnetic resonance imaging

**DOI:** 10.6061/clinics/2020/e1766

**Published:** 2020-08-21

**Authors:** Jamilly Gomes Maciel, Iana Mizumukai de Araújo, Lucio C. Trazzi, Paulo Mazzoncini de Azevedo-Marques, Carlos Ernesto Garrido Salmon, Francisco José Albuquerque de Paula, Marcello Henrique Nogueira-Barbosa

**Affiliations:** I Departamento de Imagens Medicas, Hematologia e Oncologia Clinica, Faculdade de Medicina de Ribeirao Preto (FMRP), Universidade de Sao Paulo, Ribeirao Preto, SP, BR.; II Medicina Interna, Faculdade de Medicina de Ribeirao Preto (FMRP), Universidade de Sao Paulo, Ribeirao Preto, SP, BR.; III Departamento de Fisica, Faculdade de Filosofia, Ciencias e Letras (FFCL), Universidade de São Paulo, Ribeirao Preto, SP, BR.

**Keywords:** Magnetic Resonance Imaging, Textural Attribute, Bone Mineral Density

## Abstract

**OBJECTIVE:**

Dual-energy X-ray absorptiometry (DXA)-derived bone mineral density (BMD) often fails to predict fragility fractures. Quantitative textural analysis using magnetic resonance imaging (MRI) may potentially yield useful radiomic features to predict fractures. We aimed to investigate the correlation between BMD and texture attributes (TAs) extracted from MRI scans and the interobserver reproducibility of the analysis.

**METHODS:**

Forty-nine volunteers underwent lumbar spine 1.5-T MRI and DXA. Three-dimensional (3-D) gray-level co-occurrence matrices were measured from routine sagittal T2 fast spin-echo images using the IBEX software. Twenty-two TAs were extracted from 3-D segmented L3 vertebrae. The estimated concordance coefficient was calculated using linear regression analysis. A Pearson correlation coefficient analysis was performed to evaluate the correlation between BMD and the TAs. Interobserver reproducibility was assessed with the concordance coefficient described by Lin.

**RESULTS:**

The results revealed a fair-to-moderate significant correlation between BMD and 13 TAs (*r*=−0.20 to 0.39; *p*<0.05). Eight TAs (autocorrelation, energy, homogeneity 1, homogeneity 1.1, maximum probability, sum average, sum variance, and inverse difference normalized) negatively correlated with BMD (*r*=−0.20 to −0.38; *p*<0.05), whereas five TAs (dissimilarity, difference entropy, entropy, sum entropy, and information measure corr 1) positively correlated with BMD (*r*=0.29-0.39; *p*<0.05). The interobserver agreement was almost perfect for all significant TAs (95% confidence interval, 0.92-1.00; *p*<0.05).

**CONCLUSION:**

Specific TAs could be reliably extracted from routine MRI and correlated with BMD. Our results encourage future evaluation of the potential usefulness of quantitative texture measurements from MRI scans for predicting fragility fractures.

## INTRODUCTION

Osteoporosis is the most common metabolic bone disease and is characterized by reduced bone mineral density (BMD), deteriorated bone microarchitecture, and increased bone fragility and susceptibility to fractures (1).

BMD quantified with dual-energy X-ray absorptiometry (DXA) is used as a major determinant of osteoporosis; however, bone mass measurements are insufficient to correctly predict all osteoporotic fragility fractures. In spite of this, DXA continues to be an important tool in the clinical evaluation of osteoporosis. Moreover, DXA is still used as an initial, but insufficient, step in the evaluation of the efficacy of new drugs. This has encouraged the development of other techniques that can reveal bone strength abnormalities. Currently, several bone quality parameters are used with clinical data to improve the prediction of future fragility fractures (2-4).

Together, bone geometry, cortical porosity, collagen properties, bone turnover rates, trabecular microarchitecture, and percentage of microdamage, and bone marrow adiposity (BMAT) are bone strength determinants. In addition, each property may independently contribute to an increased or decreased risk of fracture, even without changes in BMD (5-10). Recent studies have shown that texture-derived features extracted from multidetector computed tomography (MDCT) and DXA scans can indirectly quantify the trabecular bone microarchitecture and thus improve the traditional examination of bone quality and the prediction of fragility fractures (11,12).

The gray-level co-occurrence matrix (GLCM) model is a texture analysis method that uses several statistical parameters to quantify the spatial relationship between diagnostic image pixels, allowing characterization of the morphological properties of underlying tissues (13,14). This model has been validated for characterizing textural attributes (TAs) from MDCT images; however, a few studies have used the same model to extract TAs from magnetic resonance imaging (MRI) scans. Most of these studies extracted texture patterns from the MRI scans of patients with tumors to improve diagnostic accuracy and prognostic prediction (15-17).

MRI is a versatile imaging technique with great potential for enabling the multiparametric analysis of bone quality in a single examination. Advanced MRI sequences such as proton spectroscopy and gradient-echo sequences have been used to quantify BMAT and trabecular bone microarchitecture, respectively (18-25). However, to the best of our knowledge, no study has used routine MRI sequences to extract bone TA, a tool that can be used to indirectly study the microarchitecture of trabecular bone.

This study investigated the interobserver reproducibility of bone texture parameters extracted from routine sagittal T2-weighted fast spin-echo (FSE) lumbar spine (LS) MRI sequences obtained from healthy eutrophic individuals by using GLCM texture features and evaluated their relationship with BMD.

## ETHICAL CONSIDERATIONS

Approval by our institutional ethics committee was obtained (47580315.5.0000.5440). All the participants agreed to participate in the study and provided written informed consent. In addition, the procedures were performed in accordance with the Declaration of Helsinki.

## MATERIALS AND METHODS

### Subjects

The study subjects were comprised 49 volunteers who underwent MRI and DXA between May 2011 and October 2014. Both examinations were performed on the same day. All the individuals were at least 18 years old and had a body mass index (BMI) between 18 and 25 kg/m^2^. Individuals who used medications that affect bone metabolism, reported histories of smoking and/or alcoholism in the last 10 years, and presented any structural abnormality in the LS that could affect MRI or DXA measurements were excluded from the study.

### Dual-energy X-ray absorptiometry

The BMD of the LS (L1-L4) was determined using DXA (Hologic Discovery Wi, QDR series, Hologic, Inc., Waltham, MA, USA). The precision error in the LS was 1.2%.

### MRI and TA extraction

**Figure 1:**
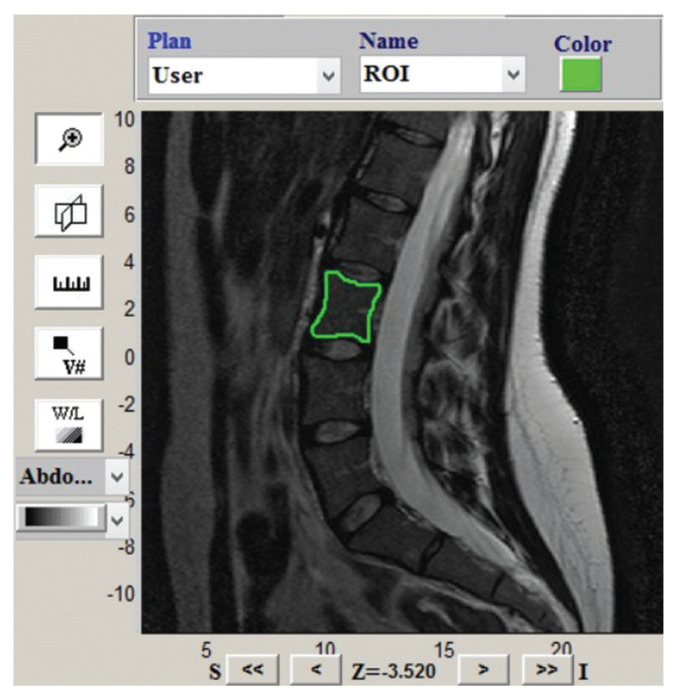
A 31-year-old female volunteer. Illustrated are the segmentation and positioning of the volume of interest (VOI) in the L3 vertebral body using the IBEX program for volumetric (three-dimensional) extraction of textural attributes (TAs).

All the subjects underwent LS MRI on a 1.5-Tesla system (Philips Achieva, Philips Medical Systems, Best, the Netherlands). Routine sagittal T2-weighted FSE sequences were used to extract TAs (echo time [TE], 120 ms; gap, 4.4 mm; echo-train length, 19; repetition time [TR], 3900 ms; slice thickness, 3.0 mm; scan duration, 2 min 16 s). The L3 vertebrae were manually segmented by two trained raters (JGM, a musculoskeletal radiologist with 3 years of experience in this area) and (LCT, a medical student research fellow). The GLCM characteristics were segmented and extracted using the radiomics analysis platform IBEX v 1.0 (26) as demonstrated in Figure 1.

The IBEX software characterizes the spatial distribution of gray-level intensities in a region (ROI) or volume of interest (VOI) (27). GLCMs obtained from a VOI or ROI are used to calculate the probability of occurrence of pixel/voxel pairs of gray-level intensities *i* and *j* given a distance *d* from an orientation *θ* (13,28).

A total of 22 attributes were identified from the L3 segmented VOI. Thus, volumetric three-dimensional (3-D) GLCM (COM3Ds) analyses were performed for the vertebral body. COM3Ds directly computed the occurrence of intensity pairs in the orientation *θ* for the dimensions *x* and *y*, and in the orientation *φ* for the dimension *z*. The distances ranged from 1 to 5 voxels, and all 13 orientations of *θ* and *φ* (*θ*=0° and *φ*=0°, 45°, 90°, and 135°; *θ*=45° and *φ*=45°, 90°, and 135°; *θ*=90° and *φ*=45°, 90°, and 135°; and *θ*=135° and *φ*=45°, 90°, and 135°) were used, resulting in 65 COM3Ds. The 22 GLCM attributes extracted were autocorrelation, cluster prominence, cluster shade, cluster tendency, contrast, correlation, difference entropy, dissimilarity, energy, entropy, two measures of homogeneity (homogeneities 1 and 2), two measures of information correlation (IMC1 and IMC2), inverse difference moment normalized, inverse difference normalized, inverse variance, maximum probability, sum average, sum entropy, sum variance, and variance.

### Statistical analyses

The results were analyzed using one-way analysis of variance followed by the Duncan posttest. The confidence interval (CI) was 95%, and the level of significance was set at 0.05. Simple (model 1) and multiple (model 2) linear regression models were tested to verify the association of the parameters. Model 2 was adjusted by age and BMI. In addition, the Pearson correlation coefficient (*r*) was calculated to measure the strength of the relationship between the variables. The interobserver agreement was evaluated using the concordance test proposed by Lin in 1989 (29). The levels of strength of the agreement associated with kappa statistics were described according to the corresponding kappa ranges as follows: poor, 0.00-0.19; fair, 0.20-0.39; moderate, 0.40-0.59; substantial, 0.60-0.79; and almost perfect, 0.80-1.00 (30). All analyses were performed using the SAS 9.4 software (31).

## RESULTS

### Patients’ demographics, clinical characteristics, and BMD

The study subjects were comprised 49 healthy individuals with a mean age of 41±14.2 years (range, 20-68 years; median, 39 years) and a mean BMI of 22.6 kg/m^2^. Of the subjects, 20 were men (mean age, 41±15.5 years; range, 20-68 years) and 29 were women (mean age, 40.9±13.4 years; range, 21-66 years). All nine women aged >50 years were in the postmenopausal stage. The mean BMI was similar between the men and the women (Table 1). The LS BMD results were available for all the subjects. No significant difference in BMD measured with DXA was found between the men and the women. In addition, the differences in the BMDs of the LS (L1-L4) and third lumbar vertebral body (L3) were not significant (*p*>0.05; Table 1).

### Correlation between BMD and bone TAs

The results revealed a fair-to-moderate significant correlation between BMD and 13 TAs derived from MRI scans (*r*=−0.20 to 0.39; *p*<0.05). Eight TAs (autocorrelation, energy, homogeneity 1, homogeneity 1.1, maximum probability, sum average, sum variance, and inverse difference normalized) negatively correlated with BMD (*r*=−0.20 to −0.38; *p*<0.05), and five TAs (dissimilarity, difference entropy, entropy, sum entropy, and information measure corr 1) positively correlated with BMD (*r*=0.29-0.39; *p*<0.05; Table 2).

### Inter-rater reliability

This study evaluated the agreement between two observers (JGM and LCT) for the 13 significant TAs derived from MRI scans. Both researchers performed manual segmentation of the L3 vertebra using the IBEX software and extracted TAs using the software. Both researchers were blinded to the patients’ clinical data and identification. The extracted values were tabulated in Excel, and the agreement coefficient was calculated using the Lin coefficient. The results demonstrated an almost perfect interobserver agreement for all 13 significant TAs extracted (95% CI, 0.92-1.00; *p*<0.05). The level of agreement of the two observers was greater for the following parameters: autocorrelation, cluster tendency, contrast, difference entropy, dissimilarity, energy, entropy, homogeneity 1, homogeneity 2, inverse different moment normalized, inverse different normalized, inverse variance, maximum probability, sum average, sum entropy, sum variance, and variance.

## DISCUSSION

Radiomics is a research area that has shown promising growth and usefulness in diagnostic and prognostic evaluations and therapeutic decision making (32-34). Texture-based quantitative features are used to characterize uniformity, randomness, and repetitive patterns in an image and have been used to detect pathologies and malignancies. Moreover, texture analysis using spectral and structural attributes extracted from MRI scans has been used to differentiate between osteoporotic and malignant compression fractures of vertebral bodies, with promising results reported in the literature (35).

Texture features have been used to study the microarchitectural characteristics of the trabecular bone from high-resolution quantitative computed tomography (HR-pQCT) images. However, no studies have been published using GLCM to extract TAs that may reflect the microarchitecture of the trabecular bone and bone fragility from spinal MRI scans. In our study, we used GLCM to characterize the spatial distribution of the gray-level within volumetric LS images from routine sagittal T2-weighted MRI scans. The choice of sagittal T2-weighted FSE sequences is justified because this sequence is widely available in many routine spinal MRI protocols. The distribution of gray levels is directly impacted by the spatial resolution of MRI sequences. The same attribute is expected to present different values for the same image obtained at different spatial resolutions. In fact, a reported methodology takes advantage of this possible variation and pattern recognition techniques, using attributes extracted from a pyramid of images taken from the same original image at different spatial resolutions. The extent of the effect of this variation on the results depends on the content of the image. We used standardized acquisitions to avoid significant variations in attribute values due to the acquisition parameters.

In this work, the two observers showed good agreement for all TAs extracted from routine MRI, and we found a significant correlation between BMD and 13 TAs. Our results suggest that specific texture parameters could be reliably extracted from routine MRI scans, with clinically acceptable reproducibility for most of the parameters studied.

Our results should encourage future studies to identify isolated attributes or a set of attributes to possibly differentiate subjects at risk of fragility fractures when used with clinical data and other bone quality parameters. Quantitative measurements based on TAs could potentially discriminate subjects with bone mass loss and those at risk of fragility fractures.

This study has some limitations that deserve mention. The cervical and thoracic spinal segments were not studied. This is a relative limitation because currently, only the lumbar vertebrae are used to measure BMD. In addition, our study population was mainly composed of young volunteers with normal bone mass and without fragility fractures. Finally, our study used BMD as a surrogate parameter of fracture susceptibility and did not test the potential usefulness of textural features extracted from MRI scans to directly predict fracture risk. However, our study provides important preliminary data for a more comprehensive study to examine the role of the technique in the evaluation of fracture susceptibility. Future studies are necessary to evaluate the potential clinical application of texture analysis using spine MRI.

## CONCLUSION

In summary, several specific TAs could be reliably extracted from routine sagittal T2-weighted MRI scans and showed a strong relationship with BMD in a healthy adult eutrophic population. Our results encourage future evaluations of MRI quantitative textural measurements to potentially discriminate subjects at risk of fragility fractures.

## AUTHOR CONTRIBUTIONS

Maciel JG performed the experiments, analyzed the data, wrote the manuscript, and reviewed the manuscript. Araújo IM analyzed the data and reviewed/edited the manuscript. Trazzi LC analyzed the data and reviewed/edited the manuscript. Azevedo‐Marques PM analyzed the data and reviewed/edited the manuscript. Salmon CEG analyzed the data and reviewed/edited the manuscript. Paula FJA analyzed the data and reviewed/edited the manuscript. Nogueira‐Barbosa MH designed the study and reviewed/edited the manuscript.

## Figures and Tables

**Table 1:** Demographics, clinical characteristics, and bone mineral density of the study participants.

	Female (n=29)	Male (n=20)	*p-*value
Age (years) (Mean±SD)	40.9±13.4	41±15.5	0.98
BMI (kg/m^2^) (Mean±SD)	22.3±2.6	23.0±1.9	0.30
Bone mineral density (BMD)			
L1-L4 BMD (g/cm^2^) (Mean±SD)	0.99±0.11	1.00±0.15	0.88
L3 BMD (g/cm^2^) (Mean±SD)	1.03±0.11	1.02±0.16	0.82

**Table 2:** Estimated coefficients of the association between bone mineral density and bone texture attributes.

Bone mineral density (g/cm^2^)			
Texture attribute	Estimated coefficient	*p*-value	Pearson correlation coefficient (*r*)
Autocorrelation	−0.0000046	0.0023	0.32
Difference entropy	0.076	0.0087	−0.29
Dissimilarity	0.0079	0.0485	−0.20
Energy	−0.62	0.0070	0.33
Entropy	0.028	0.0024	−0.38
Homogeneity 1	−0.47	0.0020	0.38
Homogeneity 2	−0.44	0.0020	0.39
Information measure corr 1	2.16	0.0281	−0.35
Inverse different normalized	−2.73	0.0308	0.22
Max probability	−0.42	0.0021	0.39
Sum average	−0.00083	0.0038	0.29
Sum entropy	0.053	0.0086	−0.31
Sum variance	−0.0000012	0.0022	0.32
